# Bench-test comparison of 26 emergency and transport ventilators

**DOI:** 10.1186/s13054-014-0506-0

**Published:** 2014-10-15

**Authors:** Erwan L’Her, Annie Roy, Nicolas Marjanovic

**Affiliations:** Chaire de Recherche en Médecine d’Urgence, Université Laval/Centre hospitalier affilié universitaire de Lévis, Québec, Canada; Département de Médecine Familiale et Médecine d’Urgence, Université Laval, Québec, Canada; Centre de Recherche du Centre hospitalier affilié universitaire de Lévis et CHA Enfant-Jésus, Québec, Canada; Pôle Urgences-SAMU, CHU de la Cavale Blanche, 29609 Brest Cedex, France; Centre de Simulation en Santé, LATIM, INSERM UMR 1101, Université de Bretagne Occidentale, 29200 Brest, France; Réanimation Médicale et Urgences Adultes, CHU de la Cavale Blanche, Brest Cedex, France

## Abstract

**Introduction:**

Numerous emergency and transport ventilators are commercialized and new generations arise constantly. The aim of this study was to evaluate a large panel of ventilators to allow clinicians to choose a device, taking into account their specificities of use.

**Methods:**

This experimental bench-test took into account general characteristics and technical performances. Performances were assessed under different levels of FIO_2_ (100%, 50% or Air-Mix), respiratory mechanics (compliance 30,70,120 mL/cmH_2_O; resistance 5,10,20 cmH_2_O/mL/s), and levels of leaks (3.5 to 12.5 L/min), using a test lung.

**Results:**

In total 26 emergency and transport ventilators were analyzed and classified into four categories (ICU-like, n = 5; Sophisticated, n = 10; Simple, n = 9; Mass-casualty and military, n = 2). Oxygen consumption (7.1 to 15.8 L/min at F_I_O_2_ 100%) and the Air-Mix mode (F_I_O_2_ 45 to 86%) differed from one device to the other. Triggering performance was heterogeneous, but several sophisticated ventilators depicted triggering capabilities as efficient as ICU-like ventilators. Pressurization was not adequate for all devices. At baseline, all the ventilators were able to synchronize, but with variations among respiratory conditions. Leak compensation in most ICU-like and 4/10 sophisticated devices was able to correct at least partially for system leaks, but with variations among ventilators.

**Conclusion:**

Major differences were observed between devices and categories, either in terms of general characteristics or technical reliability, across the spectrum of operation. Huge variability of tidal volume delivery with some devices in response to modifications in respiratory mechanics and F_I_O_2_ should make clinicians question their use in the clinical setting.

**Electronic supplementary material:**

The online version of this article (doi:10.1186/s13054-014-0506-0) contains supplementary material, which is available to authorized users.

## Introduction

Transportation of critically ill patients requiring mechanical ventilation requires clinical experience, an efficient device, and careful determination of gases [1] and/or electrical resources [2]. Emergency and transport ventilators (ETV) should be technically accurate, and as autonomous as possible.

ETV have become increasingly sophisticated, offering features that were once reserved for the ICU. Noninvasive ventilation (NIV) has become standard during acute respiratory failure in the emergency department (ED) [3] and is now available in most ETV. However, it requires synchronization of ventilator cycling to respiratory muscles activity, in order to be effective [4–6] and well-tolerated [7]. While NIV availability seems necessary for devices dedicated to acute care [8], it may not be mandatory for transportation solely. A good triggering performance is also mandatory while deep sedation is to be avoided to decrease ventilatory assistance duration.

To our knowledge, while several studies have focused on ETV [9–13], most of them only investigated a few devices. The aims of this study were to provide an objective evaluation of the widest panel of ETV, available in Europe and Northern America. We also aimed to draw standards for an objective choice, taking into account clinicians’ specificities of use.

## Materials and methods

### General characteristics

General characteristics (volume, weight, size, noise) were recorded using standardized techniques [14]. Duration of operation from a battery (electrical autonomy) was assessed after two battery charge/discharge cycles, from ventilation initiation to cessation. Gas consumption was evaluated using a filled oxygen cylinder, until effective ventilation cessation. Several devices do not enable precise oxygen inspiratory fraction (FIO_2_) setting, but use an Air-Mix, which produces a specific air-mixing condition using the Venturi effect_._ The Venturi device within the ventilator is used to entrain a fixed amount of ambient air into a high-pressure oxygen supply, thus resulting in variable FIO_2_ and gas flow. All autonomy sessions were performed under standardized settings as follows: tidal volume (VT) = 500 mL; respiratory rate (RR) = 15 breaths (b)/minute; FIO_2_ = 50%/Air-Mix and 100%.

### Technical performance

Performance was assessed under three levels of FIO_2_ (100%, 50% or Air-Mix, 21%) and respiratory mechanics combinations as follows: compliance (C) = 30, 70, 120 mL/cm H_2_O; resistance (R) = 5, 10, 20 cm H_2_O/mL/s. All measures were performed at atmospheric pressure, constant room temperature (22°C) and test-lung temperature (37°C), using an ASL5000™ lung simulator (Ingmar, Pittsburgh, PA, USA) [14,15].

#### Volume delivery and pressurization accuracy

Volume-controlled ventilation reliability was evaluated at VT = 500 mL, RR = 12 b/min, PEEP = 5 and 10 cm H_2_O, without inspiratory effort. Pressurization accuracy assessed PEEP stability (PEEP = 10 cm H_2_O) in continuous positive airway pressure (CPAP) mode, and inspiratory pressure (pressure support (PS) = 10 cm H_2_O) in the pressure support mode (PSV). For spontaneous breathing, the intensity of effort was quantified by the pressure decrease at 0.1 s, and a single low-effort value was chosen (P0.1 = 2 cm H_2_O). A 10% variability value for all these parameters was a priori considered clinically relevant for reliability.

#### Triggering evaluation

Experimental conditions reproduced those previously reported [16–18], and effort intensity was also set at P0.1 = 2 cm H_2_O. Triggering performance was assessed according to the following criteria: 1) triggering delay (DT) between onset of the airway pressure decay and flow delivery; 2) pressurization delay, which is the time at which the airway pressure signal rose (DP), and 3) airway pressure-time product per cycle (PTP) during the trigger phase, defined as the area under the Paw signal during the DT interval. Overall inspiratory delay (DI) is composed of these two components (DT + DP), and a shorter DI indicates better triggering performance.

#### Asynchrony management

PSV was delivered at similar respiratory mechanics and P0.1 values, under three levels of circuit leaks ranging from 3.5 to 4.0 (L1), 5.0 to 7.0 (L2) and 9.0 to 12.5 L/minute (L3). Leaks were applied using the dedicated ASL5000 module. Asynchrony index (AI) was calculated over a 1-minute period, after signal stabilization, and took into consideration all major types of asynchrony: failed triggering, auto-triggering, prolonged inspiration, multiple triggering, premature and short cycling. Measurements were performed under factory settings for inspiratory time and expiratory trigger without the NIV mode, and then under the NIV mode when available. AI ≥10% of respiratory effort was considered clinically significant [19].

### Tested devices

Twenty-six ETV were compared and a priori classified according to four categories (Tables 1 and 2), taking into account manufacturers’ presentation of their devices. ICU-like ETV are devices that, even if transportable, cannot be considered for transportation on a routine basis; sophisticated ETV usually depict curve monitoring screens and allow noninvasive ventilation; simple ETV are standard devices providing no extensive monitoring; mass-casualty/military ETV are devices dedicated for field operations. They are quite heavy and depict little monitoring, but are very robust and may run without oxygen availability.

**Table 1:** **Emergency and transport ventilators’ general characteristics (ICU-like and sophisticated)**

**Device**	**Company**	**Technology**	**Pediatric use (≥10 kg)**	**FIO**_**2**_ **(range/%)**	**Circuit**	**Built-in PEEP**	**Weight (kg)**	**Volume (dm** ^**3**^ **)**	**Screen (with curves)**	**Sonometry function (min/max, dB)**	**Sonometry alarm (max, dB)**	**Oxygen consumption FIO**_**2**_ **100%/50% or Air-Mix (L/min)**	**Maximal autonomy FIO**_**2**_ **100% cylinder E (min)**	**Electrical autonomy (min)**	**Market price ($ US)**
**ICU-like emergency and transport ventilators** (n = 5)															
**Bellavista**	IMT Medical (Buchs, Switzerland)	Turbine	Yes	100%/21%	Mono	Yes	7.9	25.2	Yes	ND	ND	13.3/ND	51	75	20,000 to 25,000 US $
**C2**	Hamilton Medical (Bonaduz, Switzerland)	Turbine	Yes	100%/21%	Mono/dual	Yes	9.6	29.0	Yes	50.4/50.6	63.2	15.8/3.3	43	142	20,000 to 25,000 US $
**Servo-i**	Maquet Getinge (Göteborg, Sweden)	Pneumatic	Yes	100%/21%	Dual	Yes	20.4	39.0	Yes	42.1/42.6	64.6	8.4/4.3	81	146	>25,000 US $
**T75**	Air Liquide Medical Systems (Antony, France)	Turbine	Yes	100%/21%	Mono/dual	Yes	16.0	38.4	Yes	48.1/50.5	75.7	11.1/3.5	61	200	20,000 to 25,000 US $
**Vela**	Viasys Carefusion (San Diego, Ca, USA)	Turbine	Yes	100%/21%	Dual	Yes	17.3	28.6	Yes	46.5/51.5	79.1	15.5/6.3	44	324	20,000 to 25,000 US $
**Sophisticated emergency and transport ventilators** (n = 10)															
**Crossvent 3**	Bio-Med Devices (Guilford, CT, USA)	Pneumatic	Yes	100%/21%	Mono	Yes	4.8	6.7	Yes	42.7/52.4	76.8	8.3/4.5	82	429	10,000 to 15,000 US $
**Elisée**	Resmed (Saint Priest, France)	Turbine	Yes	100%/21%	Mono/dual	Yes	4.7	9.5	Yes	47.4/52.3	78	15/3.5	45	196	10,000 to 15,000 US $
**HT 50**	Newport (Costa Mesa, CA, USA)	Piston	Yes	100%/21%	Mono/dual	Yes	8.3	16.6	No	42.9/48	46.4	7.4/2.9	92	> 840	10,000 to 15,000 US $
**HT 70 plus**	Newport Covidien (Mansfield, MA, USA)	Piston	Yes	100%/21%	Mono/dual	Yes	6.9	23.7	No	43.8/64.7	71.8	7.7/4.3	88	488	15,000 to 20,000 US $
**iVent 201**	GE Healthcare (Cleveland, OH, USA)	Turbine	Yes	100%/21%	Mono	Yes	11.4	19.0	Yes	51.0/56.2	88.5	8.9/2.6	76	ND	20,000 to 25,000 US $
**LTV 1200**	Viasys Carefusion (San Diego, Ca, USA)	Turbine	Yes	100%/21%	Mono	Yes	6.3	5.7	No	54.3/61.1	80	12.6/4.3	54	127	10,000 to 15,000 US $
**M. Transport**	Weinmann (Hamburg, Germany)	Pneumatic	Yes	100%/40%	Mono	Yes	4.8	7.0	Yes	46.6/51.2	56.7	7.1/2.4	96	365	15,000 to 20,000 US $
**Oxylog 3000**	Dräger (Lübeck, Germany)	Pneumatic	Yes	100%/40%	Mono	Yes	5.0	8.5	Yes	37/52.7	49.6	7.1/2.8	96	483	15,000 to 20,000 US $
**T 1**	Hamilton Medical (Bonaduz, Switzerland)	Turbine	Yes	100%/21%	Mono/dual	Yes	6.5	19.7	Yes	ND	88	10.3/3.4	66	408	20,000 to 25,000 US $
**T 60**	Air Liquide Medical Systems (Antony, France)	Turbine	Yes	100%/21%	Mono/dual	Yes	4.0	8.0	Yes	ND	79	11.1/3.8	61	384	15,000 to 20,000 US $

Pediatric use, availability of a pediatric mode; FIO_2_, oxygen inspiratory fraction (%); Circuit, type of circuit to be used, monobranched, dual limb, both types; PEEP, positive end expiratory pressure; Volume, the volume of the device was calculated as assimilated to a cube; Sonometry, provides the noise level in function - values are provided without added PEEP and for FIO_2_ = 100% for most values, for FIO_2_ = 100%/50% for the two other; Autonomy was evaluated at standardized settings - MCV 100 electrical autonomy has been calculated while using the compressor; Market price has been considered taking into account mean value provided by various distributors on the Canadian and the European market. The air-mixing condition varies from one ventilator to the other (FIO_2_ = 45 to 86%), but also for the same ventilator, according to the patient’s condition (FIO_2_ AirMix Osiris = 69 to 81%; O2000 = 68 to 80%); it may induce up to a 30% peak-flow decrease with several devices; this flow decrease at a constant I/E ratio is responsible for a tidal volume decrease (FIO_2_ or Venturi effect). ND= not done.

**Table 2:** **Emergency and transport ventilators’ general characteristics (simple and mass-casualty/military)**

**Device**	**Company**	**Technology**	**Pediatric use (≥10 kg)**	**FIO**_**2**_ **(range/%)**	**Circuit**	**Built-in PEEP**	**Weight (kg)**	**Volume (dm** ^**3**^ **)**	**Screen (with curves)**	**Sonometry function (min/max, dB)**	**Sonometry alarm (max, dB)**	**Oxygen consumption FIO**_**2**_ **100%/50% or Air-Mix (L/min)**	**Maximal autonomy FIO**_**2**_ **100% cylinder E (min)**	**Electrical autonomy (min)**	**Market price ($ US)**
**Simple emergency and transport ventilators** (n = 9)															
**Autovent 3000**	Allied Healthcare Products (Saint Louis, MO, USA)	Pneumatic	Yes	100% only	Mono	No	2.8	4.8	No	47.2/48.0	55.3	9.7/NA	70	NA	5,000 to 10,000 US $
**Carevent MRI**	O-Two (Mississauga, ON, Canada)	Pneumatic	Yes	100%/Air-Mix	Mono	Yes	2.5	5.4	No	43.2/52.7	78.7	10.0/5.2	68	NA	5,000 to 10,000 US $
**M. Standard**	Weinmann (Hamburg, Germany)	Pneumatic	Yes	100%/Air-Mix	Mono	No	1.2	4.0	No	42.9/44.3	49.8	8.5/3.5	80	NA	5,000 to 10,000 US $
**Osiris 2**	Air Liquide Medical Systems (Antony, France)	Pneumatic	Yes	100%/Air-Mix	Mono	Yes	5.0	9.9	No	47.3/53.3	83.0	11.4/5.3	56	NA	5,000 to 10,000 US $
**Oxylog 2000**	Dräger (Lübeck, Germany)	Pneumatic	Yes	100%/Air-Mix	Mono	No	4.3	5.3	No	46.6/61.6	78.8	9.4/4.7	72	ND	5,000 to 10,000 US $
**Oxylog 2000 +**	Dräger	Pneumatic	Yes	100%/Air-Mix	Mono	Yes	5.4	8.4	Yes	46.2/52.2	77.0	7.7/2.1	88	ND	10,000 to 15,000 US $
**ParaPAC 210 D**	Smiths Medical (Ashford, UK)	Pneumatic	Yes	100%/Air-Mix	Mono	No	3.2	3.0	No	43.9/45.6	56.6	8.8 /3.3	77	NA	2,500 to 5,000 US $
**ParaPAC 310**	Smiths Medical (Ashford, UK)	Pneumatic	Yes	100%/Air-Mix	Mono	Yes	2.3	3.6	No	ND	ND	ND	ND	NA	2,500 to 5,000 US $
**pNeuton**	Airon (Melbourne, FL, USA)	Pneumatic	No	100%/Air-Mix	Mono	Yes	3.5	7.9	No	43.4/61.3	84.9	11.1/NA	61	NA	5,000 to 10,000 US $
**Mass-casualty and military ventilators** (n = 2)															
**ComPAC**	Smiths Medical (Ashford, UK)	Pneumatic/compressor	No	100% O_2_/Air-Mix or 100% air	Mono	No	11.1	18.0	No	41.7/65.8	42.0	7.1/2.4	95	78	5,000 to 10,000 US $
**MCV100**	Allied Healthcare Products (Saint Louis, MO, USA)	Pneumatic/compressor	No	Only 100% O_2_ or 100% air	Mono	No	6.9	9.0	No	43.0/48.3	61.8	7.1/NA	96	298/1,142	5,000 to 10,000 US $

Pediatric use, availability of a pediatric mode; FIO_2_, oxygen inspiratory fraction (%); Circuit, type of circuit to be used, monobranched, dual limb, both types; PEEP, positive end expiratory pressure; Volume, the volume of the device was calculated as assimilated to a cube; Sonometry, provides the noise level in function - values are provided without added PEEP and for FIO_2_ = 100% for most values, for FIO_2_ = 100%/50% for the two other; Autonomy was evaluated at standardized settings - MCV 100 electrical autonomy has been calculated while using the compressor; Market price has been considered taking into account mean value provided by various distributors on the Canadian and the European market. The air-mixing condition varies from one ventilator to the other (FIO_2_ = 45 to 86%), but also for the same ventilator, according to patient’s condition (FIO_2_ AirMix Osiris = 69 to 81%; O2000 = 68 to 80%); it may induce up to a 30% peak-flow decrease with several devices; this flow decrease at a constant I/E ratio is responsible for a tidal volume decrease (FIO_2_ or Venturi effect). NA= not applicable; ND= not done.

All ETV were provided free of charge by the manufacturers except three (iVent201™, Crossvent3 + ™, and HT50™) according to manufacturers’ disagreement to enter the evaluation.

### Statistical analysis

Parameters were calculated from ≥20 breaths and are given as mean ± SD unless specified otherwise. When adequate, data were compared using analysis of variance (ANOVA) for repeated measures, and the nonparametric Friedman and Wilcoxon ranked tests. A *P*-value ≤0.05 was considered statistically significant. Differences ≥10% were considered clinically significant. Statistical analysis was performed using MedCalc12.7.4 (Ostend, Belgium). For more details about material and methods of measurements, see Additional file 1.

## Results

### General characteristics

ETV characteristics are depicted within Tables 1 and 2. All 15 ETV within the ICU-like and sophisticated categories (Table 1) had built-in PEEP valves, and most of them depicted ventilatory curves on a screen. There were 9/15 (60%) using a turbine and 2 using a piston to pressurize gases, which means that they were autonomous from medical gases, but dependent on electrical power; 4/15 (27%) within these categories were pneumatic devices (gas-powered), but they were also dependent on electrical power. Two ETV did not allow FIO_2_ to decrease below 40%. Other differences in these categories were depicted in terms of oxygen and electrical autonomy, and double-circuit ventilation availability.

All ventilators within the simple and mass-casualty/military ETV categories (Table 2) solely enabled the use of monobranched circuits, and only 5/11 had built-in PEEP valves. None enabled FIO_2_ variations except an Air-Mix condition, which resulted in different FIO_2_ from one device to the other (FIO_2_ = 45 to 86%) but also while using a single ETV according to settings and respiratory mechanics. One device only proposed FIO_2_ = 100%. The mass-casualty/military ETV were dependent on electrical power in the compressor mode, but could use solely compressed gases in case of electrical failure. The volume and market price of these devices was usually lower than that of the two first categories.

### Reliability of tidal volume and PEEP delivery

#### Variability according to respiratory mechanics

**Figure 1:**
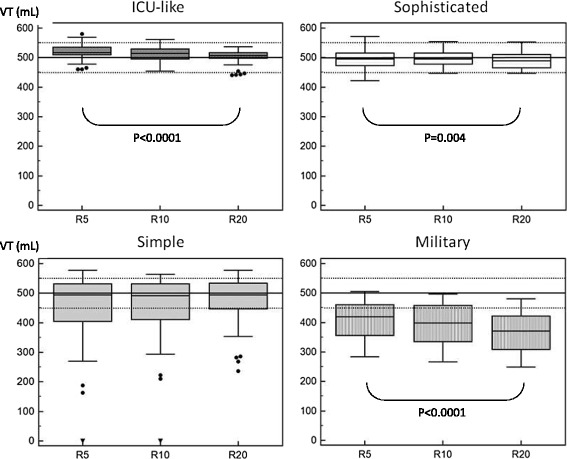
**Box plot of tidal volume according to respiratory mechanics variations in the different categories of emergency and transport ventilators (ETV).** VT, tidal volume; R, different values of resistance were applied (5, 10 and 20 cm H_2_O/L/s) in combination with different compliance (30, 70, 120 cm H_2_O/L); dotted line represents the 10% VT accuracy range; values are provided as mean (95% CI) ± STD; a *P* value equal to or below 0.05 was considered significant. ICU-like emergency and transport ventilators (ETV; n = 5) are devices that, even if transportable, cannot be considered for transportation on a routine basis; sophisticated ETV (n = 10) usually depicts curves monitoring screens and allow noninvasive ventilation; simple ETV (n = 9) are standard devices, providing no extensive monitoring; mass-casualty/military ETV (n = 2) are devices dedicated for field operations. They are quite heavy, depict little monitoring, but are very robust and may run without oxygen availability. Significant VT variations according to respiratory mechanics changes were observed for all categories, except simple ETV; all ventilators in the ICU-like and sophisticated categories were within the 10% accuracy range for VT, whereas most in the simple and in the mass-casualty/military category were outside the range.

All ETV in the ICU-like and sophisticated categories were within the accuracy range for VT, whatever respiratory mechanics combinations, while major deviations were observed in the other categories (Figure 1).

**Figure 2:**
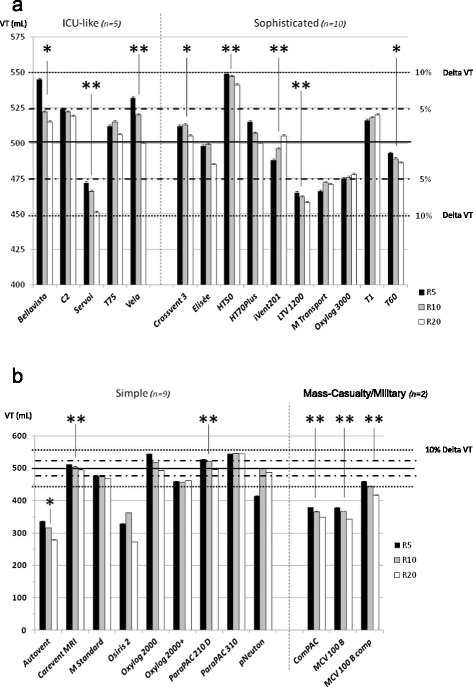
**Individual tidal volume delivery according to respiratory mechanics changes.** Values are provided as mean ± STD; VT, tidal volume; R, different values of resistance were applied (5, 10 and 20 cm H_2_O/L/s) in combination with different compliance (30, 70, 120 cm H_2_O/L); dotted line represents the 10% accuracy range and the hashed line the 5% accuracy range; a *P*-value equal to or below 0.05 was considered significant; *P <0.05; **P <0.005. **(a)** All ventilators in the ICU-like and sophisticated emergency and transport ventilator (ETV) categories were within the 10% accuracy range for VT, and most of them within the 5% accuracy range; 8 of 15 ETV depicted an impact of respiratory mechanics changes over VT accuracy; **(b)** 6 of 11 ventilators in the simple and mass-casualty/military categories developed VT below the 10% accuracy range, and 6 of them depicted an impact of respiratory mechanics changes over VT accuracy. However, for most of them, the increase in resistance, combined with high compliance, was responsible of a pressure-release safety valve opening (major overdistension related to a low expiratory flow), which meant that they were not able to deliver ventilation.

Respiratory mechanics influenced VT delivery in all categories (Figure 1) and for most devices (Figure 2a), except for the simple ETV, but with huge variations between devices.

Of the simple and mass-casualty/military ETV, 6/11 did not provide VT within the 10% accuracy range (Figure 2b), even for normal respiratory mechanics.

#### Variability according to FIO_2_

**Figure 3:**
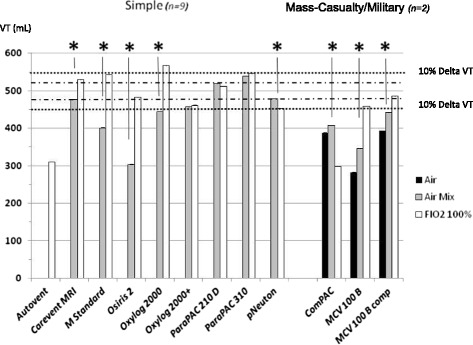
**Tidal volume accuracy according to oxygen inspiratory fraction (FIO**_**2**_ **) variations.** Values are provided as mean ± STD; VT, tidal volume; R, different values of resistance were applied (5, 10 and 20 cm H_2_O/L/s) in combination with different compliance (30, 70, 120 cm H_2_O/L); dotted line represents the 10% accuracy range and the hashed line the 5% accuracy range; for VT variations according to R, a *P*-value equal to or below 0.05 was considered significant; *P <0.05; **P <0.005. VT accuracy according to FIO_2_ variations was correct for all ICU-like and sophisticated emergency and transport ventilators (ETV). However, most simple and mass casualty/military ETV depicted a significant Venturi effect with a decrease in VT while switching from FIO_2_ 100% to Air-Mix. Moreover, clinicians might be aware that the FIO_2_ value in the Air-Mix setting greatly differed from one device to the other (from 45 to 86%).

No effect was observed in the ICU-like and sophisticated ETV categories, while in the simple and mass-casualty/military categories it induced significant impact on VT for most devices (Figure 3).

#### PEEP delivery

**Figure 4:**
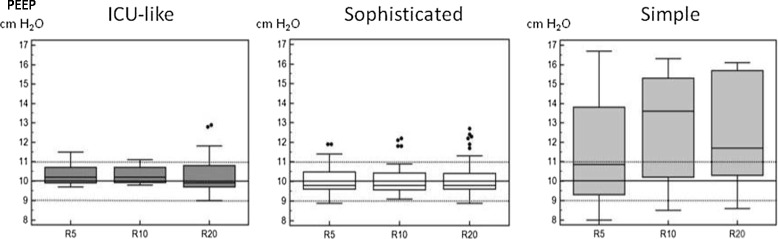
**Box plot of positive end-expiratory pressure (PEEP) values according to respiratory mechanics changes in the different categories.** R, different values of resistance were applied (5, 10 and 20 cm H_2_O/L/s) in combination with different compliance (30, 70, 120 cm H_2_O/L); dotted line represents the 10% accuracy range and the hashed line the 5% accuracy range; values are provided as mean (95% CI) ± STD. Respiratory mechanics changes did not influence PEEP accuracy among categories; PEEP administration could be considered as inaccurate in the simple ETV category. No PEEP valve was available for mass-casualty and military devices.

Applied PEEP was adequate for most devices in the ICU-like and sophisticated ETV categories, without impact of respiratory mechanics. A huge deviation was observed in the simple ETV category, up to 100% in several cases (Figure 4).

### Triggering evaluation

**Figure 5:**
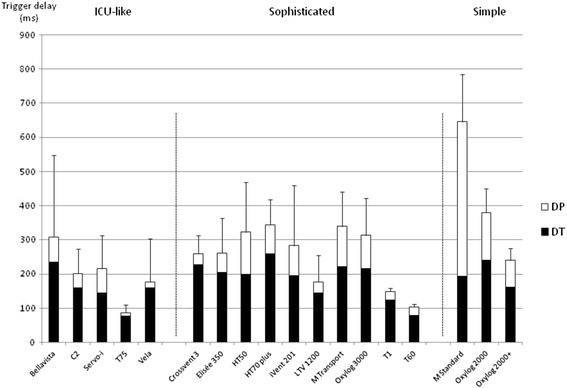
**Individual triggering values among all respiratory mechanics combinations.** Values are provided as mean ± STD; DT, triggering delay between onset of the airway pressure decay and flow delivery; DP, pressurization delay, which is the time at which the airway pressure signal rose; overall inspiratory delay (DI) is composed of these two components (DT + DP), and a shorter DI value indicates better trigger performance. Significant differences could be exhibited in terms of triggering performances while comparing one ETV category to the other.

No significant difference was observed while comparing ICU-like ETV triggering performances to sophisticated and simple ones (198 ± 80 versus 256 ± 84 versus 422 ± 206 ms respectively; *P* = 0.18) (Figure 5).

Pressure-time product per cycle (PTP) during the triggering phase tended to be higher for the simple ETV, as compared to ICU-like and sophisticated ETV (513 ± 247 versus 133 ± 119 versus 154 ± 103 cm H_2_O ms respectively; *P* = 0.08). However, few data are available for simple ETV, while ventilation was often impossible for most respiratory mechanics combinations, without any triggered ventilatory cycles, even for some devices that were presumed to allow spontaneous ventilation.

### Pressurization and NIV mode performance

**Figure 6:**
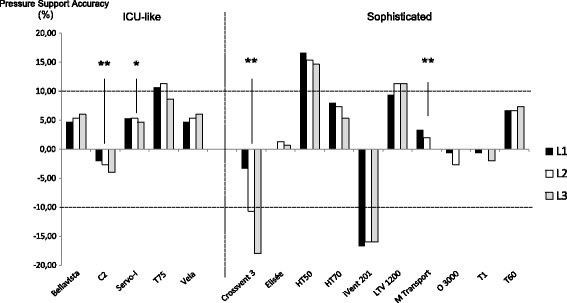
**Pressure support accuracy under different levels of leaks.** Values are provided as % difference as compared to settings (pressure support (PS) = 10 cm H_2_O with an additional positive end-expiratory pressure (PEEP) level = 5 cm H_2_O); dotted line represents the a priori defined accuracy range (±10%); three levels of leaks were used: L1 = 3.5 to 4.0 L/minute; L2 = 5.0 to 7.0 L/minute; L3 = 9.0 to 12.5 L/minute. Several devices were quite insensitive to leaks, with PS variation near zero; statistical analysis evaluated the impact of leaks over PS accuracy and a *P*-value equal to or below 0.05 was considered significant; *P <0.05; **P <0.005. Of the ETV, 10/15 provided PS within an accuracy range equal to or less than 10%. If 4/15 ETV were statistically influenced by leaks, only one might be considered as influenced with a clinical relevance, the other one being within the accuracy range.

Of the ETV, 15/26 (58%) were presumed to allow NIV, of which 4/15 (27%) did not provide PS reliability, whatever respiratory mechanics and leaks (Figure 6).

**Figure 7:**
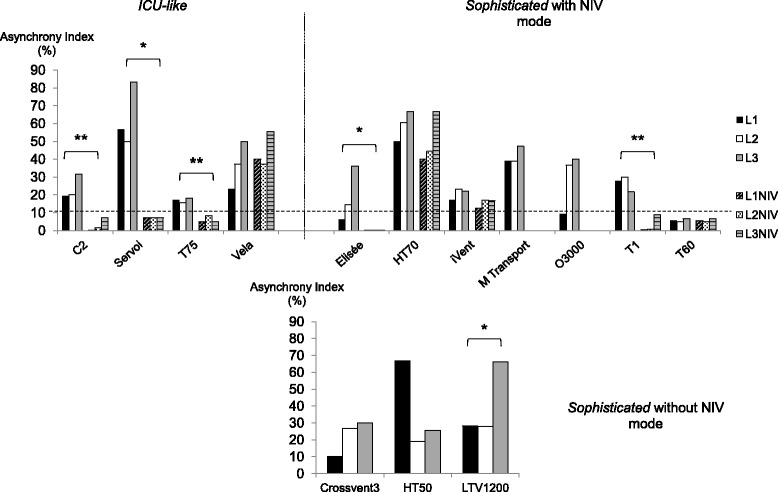
**Asynchrony management under different levels of leaks, with/without the noninvasive ventilation (NIV) mode.** Asynchrony index (AI) values are provided as% of respiratory efforts under pressure support (PS) ventilation (PS = 10 cm H_2_O + positive end-expiratory pressure (PEEP) = 5 cm H_2_O); dotted line represents the AI clinical level of significance (10%). Three levels of leaks were used: L1 = 3.5 to 4.0 L/minute; L2 = 5.0 to 7.0 L/minute; L3 = 9.0 to 12.5 L/minute. Measurements were performed under triggering modalities defined by the factory settings, and then under the noninvasive ventilation mode if available. Statistical analysis evaluated the impact of leaks over AI occurrence and the effect of the NIV mode; A *P*-value equal to or below 0.05 was considered significant; *P <0.05; **P <0.005. Most devices except one did not depict an AI variation according to increased leaks. The NIV mode effect was significant for most ICU-like devices, whereas results were heterogeneous for the sophisticated category. Several sophisticated devices depicted better results in terms of asynchrony management than mean ICU-like devices. Mass casualty/military devices were not tested as they do not enable spontaneous ventilation.

Leak increases did not modify the AI for ICU-like ETV, while it influenced efficiency for three sophisticated ETV (Figure 7). For all ICU-like ETV except one, the NIV mode enabled an AI decrease <10%. Huge heterogeneity of the NIV-mode effect was observed within the sophisticated ETV category.

## Discussion

This study allowed the comparison of a large panel of ETV, under a wide range of simulated clinical conditions. It depicts major differences between categories, but also between devices in a similar category, in terms of general characteristics and technical performance across the entire spectrum of operation. This objective comparison may enable clinicians to adapt their choice of devices according to the setting of the operation of the devices.

### General characteristics

Major differences were observed in terms of weight and volume, and ICU-like ETV seemed dedicated to use within ICUs and/or for intra-hospital transportation of severely ill patients, but not for standard out-of-hospital transport. The inclusion of the Servo-i in our testing can be discussed given its general characteristics (especially volumetry and weight), but it is positioned by the company as a portable ventilator and therefore falls into the ICU-like category.

There are mainly two categories of devices according to the technology they use to provide ventilation. Pneumatic ventilators use compressed oxygen and the other uses an electrically powered turbine to generate pressure. The performance of the second seems to be similar to those of ICU ventilators, but they are dependent on battery life [12]. A third, but less frequent option exists, the compressor or piston-driven ventilator, which represents 4/26 devices of our panel and these are also electrically powered. This technology can be combined with another technology to increase autonomy (mass-casualty/military).

Oxygen consumption differed between devices, which means that a calculation taking into account minute ventilation and oxygen availability within the canister might not be adequate. Simple ETV oxygen consumption was lower, but this could be related to the fact that they only allowed Air-Mix. Air-Mixing differed in terms of performance from one device to the other (FIO_2_ = 45 to 86%), but also for a single device (FIO_2_ = 68 to 80% with the Oxylog2000) according to respiratory mechanics and ventilation parameters, that is, oxygen consumption and hypoxemia correction may vary within a huge range, without any information provided to the clinician.

Battery duration is an important function of ETV, as it determines autonomy in environments where electricity is not available [2,20]. Such concerns about electrical power autonomy and battery duration mainly focus on the ICU-like and sophisticated ETV, whereas simple ones will mainly be concerned by gas consumption. As already investigated, battery duration differed within devices [2,11,13]. The American Association for Respiratory Care guidelines recommend a battery duration ≥4 hours for portable ventilators in pandemic and mass-casualty conditions [21]. To our knowledge, no guideline exists for standard ETV, but except for ICU-like ETV, two out of three devices had battery duration ≥4 hours. Several variables affect battery duration, including ventilator operating characteristics, whereas our evaluation was performed using a single setting. Turbine-driven ventilators are known to have a shorter battery duration than piston-driven ventilators, and constant-speed turbines use the most energy [22,23].

Noise is a frequent environmental pollutant in the hospital setting, especially within ED and ICUs. The World Health Organization (WHO) recommends that the background noise in hospitals should not exceed 30 decibels (dB), and that peaks during night should be <40 dB [24], but it has been demonstrated that average levels are usually higher within ICUs (60 to 70 dB; peaks ≥90 dB) [25]. Studies using polysomnography and environmental measurements to determine whether noise could explain irregular sleep patterns in ICU patients have reported that it caused 11 to 17% of arousals and awakenings [26]. Sleep disruption in the ICU is also associated with increased requirement for anxiety treatments [27] and biochemical markers of stress [28]. In our study, the average sonometry varied from 37 dB to 59 dB, thus, clearly crossing the WHO recommendation, but also the more realistic 45-dB limit chosen by neonatologists [29]. These results are similar to those obtained in bench testing of handy ventilators [14]. Noise measurements are rarely available for the ED [30], but it may also be considered that more stressful levels of noise may occur during transportation [31], especially when using helicopters.

### Tidal volume accuracy

No data are ever provided by the industry within the specification documents about the potential impact of respiratory mechanics over technical performance/accuracy towards parameters settings. When looking at the simple devices as an example, it seems interesting from the clinician point of view that the Parapac 201 which is quite a cheap device is accurate in terms of VT accuracy whatever respiratory mechanics, while *a contrario*, 40% of the set VT is not delivered with the Autovent. Moreover, and this could be considered as the main purpose of the study, such independent bench-testing really enables us to compare a large panel of devices on the same technical basis.

### Triggering evaluation

PSV is a partial ventilatory support during which substantial muscle activity remains, and the effort required to trigger the ventilator represents 10 to 30% of the breathing effort [16]. Lung-model studies of older-generation demand-valve systems reported values for DT in excess of 400 ms, even at maximal sensitivity [16]. These triggering values can be superimposed on what we observed in our study with the simple ETV. It is now considered that ICU ventilators may have a DT ≤60 ms [17,32].

The triggering performance was heterogeneous among ETV, and mean DT values tended to be higher than those obtained in other studies [12,18]. However this may be related to the application of a wider range of respiratory mechanics combination (R = 5 to 20 cm H_2_O/mL/s; C = 30 to 120 cm H_2_O). When comparing the same experimental conditions, results of ICU-like ETV do fall within range. Several sophisticated ETV depicted triggering capabilities as efficient as most ICU-like ventilators.

Patient effort to trigger (expressed as PTP) is relatively constant with increasing ventilator support. As demonstrated by Richard *et al*., a minor increase in P0.1 (2 versus 4 cm H_2_O) may result in a higher pressure fall and thus, a higher inspiratory effort [18]. Our simulation model confirms that most new generation ICU-like and sophisticated ETV induces low effort to trigger, while simple ETV (even the most recent devices that are presumed to enable CPAP), were not adequate for such a purpose.

### Pressurization and NIV modes

PSV is widely used during acute respiratory failure [33]. As observed by various authors, most ETV were able to maintain adequate PEEP and PS, even when there was a minimal to moderate leak [34]. However, clinicians may be aware that several devices within the sophisticated ETV category did not enable adequate pressurization with up to −18% PS delivery. This default was accentuated by leaks for one device, while such variations were lower and/or always within the acceptable range for the others. Leaks may increase patient effort and asynchrony from the ventilator, but the available NIV modes usually correct that deficiency, at least in recent ICU ventilators [35].

At baseline, all the ventilators were able to synchronize without failed triggering or auto-triggering. A study previously reported wide variations in terms of synchronization capability between devices [36]. Failed triggering more frequently occurred in chronic obstructive pulmonary disease (COPD) respiratory mechanics conditions and longer times were required to stabilize VTs, as compared to acute respiratory distress syndrome (ARDS) conditions. The T60 was the only ventilator to maintain synchrony in all scenarios, without any adjustment of sensitivity, inspiratory termination criteria, and/or NIV mode setting. However, the leak compensation of most ICU-like and 4/10 sophisticated ETV was able to correct partially or completely for system leak interferences, but with a wide variation among ventilators, as already depicted [37]. As the manufacturers have not revealed the exact triggering and cycling algorithms used during system leaks, it is difficult to explain discrepancies among the different studies.

### Limitations of the study

Several limitations of our study may be discussed. The primary limitation is that comparing a technically sophisticated ICU-like device that costs as much as $25,000 to another one below $10,000 could be considered as making no sense. For this sake, devices have been divided within several categories. However, as depicted at least between ICU-like and sophisticated categories, such bench testing also demonstrates that several cheaper devices do perform at least equally to more sophisticated and expensive ones. Moreover, comparison between devices within a similar category also enables us to depict important differences in terms of performance. A second limitation is that the study was performed on a mechanical model, which can never mimic all complexities of the interactions between a patient and a ventilator, raising the question of clinical relevance. The ASL5000 has two characteristics that make it different from patients and from the typical dual-chamber mechanical models: 1) it does not simulate expiratory efforts; 2) the Pmus profile in the ASL5000 is not modified by pressurization during the inspiratory phase. However, the bench simulated all possible situations and combinations that can be encountered in the clinical field. A final limitation could be that in some patients with acute respiratory failure, ventilatory efforts may be higher than that of our simulated efforts.

## Conclusion

We provided a wide evaluation of ETV ventilators on most general features and technical aspects of these devices. Clinicians should be aware of the significant differences that were found among these ventilators when choosing these important devices for initial management and transport of critically ill patients. As expected, huge heterogeneity in terms of general characteristics and performance were observed. We should be questioned about the great variability in terms of VT delivery with several specific devices and the fact that ETV performance might be greatly influenced by lung mechanics. NIV capabilities are also highly modified by leaks and their own NIV-mode efficiency. Such a bench-test comparison may also enable the industry to improve its products.

## Key messages

- Major differences can be observed between ETV devices and categories in terms of general characteristics and/or technical reliability
- Variability of VT delivery with some ETV across the spectrum of operations should raise questions from clinicians about their use
- The technical performance of most ETV is influenced by lung mechanics
- Triggering and leak compensation performance are crucial issues while performing noninvasive ventilation
- Several new-generation ETV may be considered as more efficient than ICU ventilators for most clinical situations

## Additional file

Additional file 1
**provides detailed information about material and methods.**

## Abbreviations

AI: the global asynchrony index between the simulated patient and the ventilator
Air-Mix: design of a specific air-mixing condition using the Venturi effect resulting in variable oxygen inspiratory fraction
ARDS: acute respiratory distress syndrome
COPD: chronic obstructive pulmonary disease
CPAP: continuous positive airway pressure
dB: decibel
DI: overall inspiratory delay, composed of the two trigger components (trigger delay + pressurization delay)
DP: pressurization delay, which is the time at which the airway pressure signal rose
DT: triggering delay between onset of the airway pressure decay and flow delivery
ED: emergency department
ETV: emergency and transport ventilators
FIO_2_: oxygen inspiratory fraction, which is a parameter for the neuro-muscular activation of the respiratory system, an important determinant for the work of breathing
NIV: noninvasive ventilation
P0.1: the negative airway pressure generated during the first 100 msec of an occluded inspiration
PEEP: positive end-expiratory pressure
Pmus: simulated patient’s respiratory muscle effort intensity
PS: pressure support
PSV: pressure support ventilation
PTP: the airway pressure-time product per cycle during the trigger phase
RR: respiratory rate
VT: tidal volume
WHO: World Health Organization
