# Supplementary material for: Bench-test comparison of 26 emergency and transport ventilators
Source: Crit Care. 2014 Oct 15;18(5):506. doi: 10.1186/s13054-014-0506-0 (PMC4197290; doi:10.1186/s13054-014-0506-0)
Supplement: Additional file 1 — provides detailed information about material and methods. [file 13054_2014_506_MOESM1_ESM.docx]

ONLINE REPOSITORY

**DETAILED MATERIAL-METHODS**

**General characteristics**

Volumetry was assessed using a laser distance-meter (Leica Disto A6^TM^, St Gallen, Switzerland), assimilating the device to a cube. Weight was measured using a precision electronic scale (PD750^TM^, MyWeight HD, USA). Sonometry was assessed measuring the average noise level with a factory-calibrated Class II sonometer (Digital Sound Meter Extech 407764^TM^, Waltham, MA, USA). Measures were carried out in the fast recording mode (response time=200ms), at the normal human ear frequency. The sonometer unit was set on a tripod at the center of a 37-m^3^ room, at a 1.25 m height above ground, and a 70-cm distance from the center of the device. Data were recorded continuously for a period of 10 minutes after stabilization of the signal, with dedicated acquisition software. Measures were carried out during normal function of the device, and at two levels of oxygen inspiratory fraction (F_I_O_2_ = 100% and 50%/Air-Mix). Sonometry also took into account the alarm sound level.

**Device autonomy**

Eighteen devices are dependent of electrical power for gas motion. Six simple ventilators are pneumatic devices that run solely with compressed gases. The two mass-casualty and military devices are dependent from electrical power while using the compressor mode, but can also run solely on compressed gases in case of electrical failure.

Electrical autonomy was assessed after at least 2 complete battery charge-discharge cycles, from the initiation of ventilation to effective ventilation cessation. Gas consumption was evaluated using a filled oxygen cylinder, until effective ventilation cessation or an expiratory tidal volume decrease ≤ 10%. The oxygen cylinder duration was recorded in minutes, and mean oxygen consumption of the device was calculated assuming 642 L gas availability within E-size cylinders. Measurements were performed twice for each device, under each setting.

All autonomy sessions were performed under standardized settings: tidal volume (VT) = 500 mL; respiratory rate (RR) = 15 b/min; F_I_O_2_ = 50%/Air-Mix and 100%.

**Technical performance**

Performance was assessed under three different levels of F_I_O_2_ when available (100%, 50% or Air-Mix, 21%) and respiratory mechanics combinations (compliance (C) = 30, 70, 120 mL/cmH_2_O; resistance (R) = 5, 10, 20 cmH_2_O/mL/s), resulting in 27 different conditions for each parameter. Most devices within the simple ventilators category did not proposed precise F_I_O_2_ variations below 100%, except an “air-mix” condition using the Venturi effect; this Air-Mix results in different F_I_O_2_ from one ventilator to the other, but also while using a single ventilator according to the settings and patient’s respiratory mechanics. All measures were performed at atmospheric pressure, constant room (22°C) and test-lung temperature (cylinder temperature=37°C), using an ASL 5000^TM^ computerized mechanical lung simulator (Ingmar, Pittsburgh, PA USA) (15,14).

The ASL5000 simulator is a computerized mechanical lung simulator consisting of a piston moving inside a cylinder. The lung model uses the equation of motion to control the movement of the piston. Flow and airway pressure are measured by ﬂow and pressure sensors at the entrance of the piston, and volume is obtained by integration of ﬂow over time. The user sets compliance, resistance, and the Pmus proﬁle/waveform. While several units do not allow precise F_I_O_2_ administration it was controlled using the internal ASL 5000 built-in oxygen sensor.

Ventilators were allowed to stabilize in each test condition for 3 to 5 min, after which at least two minutes of ventilation were recorded. Data acquisition was performed at 512 Hz and stored in a desktop computer. Offline measurements and curves analysis were performed on a breath-by-breath basis using LabVIEW (National Instruments, Austin, TX, USA) and the dedicated ASL 5000 acquisition software (v3.0.3.d).

***Volume delivery and pressurization accuracy***

Volume controlled ventilation consistency and reliability was evaluated at a VT = 500 mL, RR = 12 b/min, PEEP = 5 and 10 cmH_2_O, without any inspiratory effort, under each conditions.

Pressurization accuracy assessed PEEP delivery (PEEP = 10 cmH_2_O) in the CPAP mode, and inspiratory pressure (PS = 10 cmH_2_O, with an additional PEEP level = 5 cmH_2_O) in the Pressure Support mode (PSV). For spontaneous breathing (either under CPAP and PSV), the intensity of the effort was quantified by the pressure decrease at 0.1 s and a single low effort value was chosen (P0.1 = 2 cmH_2_O).

A 10% variability of all these parameters was *a priori* considered as the clinically relevant level of reliability.

***Triggering evaluation***

Experimental conditions for triggering evaluation reproduced those previously reported (16–18). The tested machine was in the same configuration as for PSV evaluation with the most sensible trigger without autotriggering, and set to flow-triggering if available. The intensity of the effort was also set to a P0.1 value = 2 cmH_2_O.

For each simulated condition, triggering performance was assessed according to the three following criteria: 1) the triggering delay (DT) between onset of the airway pressure decay and flow delivery, 2) the pressurization delay, which is the time at which the airway pressure signal rose (DP), and 3) the airway pressure–time product per cycle (PTP) during the trigger phase, defined as the area under the Paw signal during the DT interval. Overall inspiratory delay (DI) is composed of these two components (DT + DP), and a shorter DI value indicates better trigger performance.

***Asynchrony management under different levels of leaks***

To assess asynchrony management of each device, PSV was delivered at similar respiratory mechanics settings and P0.1 value as previously described, under 3 different levels of circuit leaks ranging from 3.5-4 (L1), 5-7 (L2) and 9-12.5 L/min (L3). Leaks were applied using the specific ASL 5000 bypass and leak valve module. Asynchrony index was calculated over a 1 minute period, after signal stabilization. Measurements were performed under factory settings for inspiratory time and expiratory trigger, without NIV mode, and then under the specific NIV mode when available. No further adjustments were performed for these parameters. The asynchrony index (AI) was measured and a level of asynchronized cycles equal or upper than 10% of respiratory efforts was considered clinically significant (19).

**Tested devices**

Twenty six devices were compared and classified according to four different classes (Tables 1-a and 1-b), taking into account manufacturers’ presentation of their systems: 2 of them were considered as dedicated mass-casualty and military ventilators, 9 as simple emergency and transport ventilators, 10 as sophisticated emergency and transport ventilators, and finally 5 as ICU-like transportable ventilators.

The MCV100 ^TM^ military and mass-casualty device is a dual-ventilator that can delivered volume either using a pneumatic system driven by oxygen (thus depending of gas availability), or a dedicated compressor (depending of electrical availability), in order to be able to work as autonomously as possible on the field of operation; both methods of ventilation were separately compared for this device.

All ventilators were kindly provided free of charge by the manufacturers except three (iVent 201 ^TM^, Crossvent 3+ ^TM^, HT50 ^TM^) that had to be rented according to manufacturers’ disagreement to enter the comparative test. Devices that enabled double circuit ventilation were tested using a standard disposable ventilator circuit (Intersurgical, Berkshire, UK; circuit compliance = 2 ml/cmH_2_O). Devices that solely enabled monobranched ventilation were tested using their native circuits and expiratory valves.

**Statistical analysis**

Parameter values were calculated from at least 20 breaths and are given as mean ± SD, unless specified otherwise. When adequate, data were compared using ANOVA for repeated measures, and non-parametric Friedman’s and Wilcoxon’s ranked tests. A p value equal or below 0.05 was considered statistically significant. Differences greater than 10% were considered clinically significant. Statistical analysis was performed using MedCalc 12.7.4 for Windows (MedCalc software, Ostend, Belgium).
